# Gene expression profiles and pathway enrichment analysis of human osteosarcoma cells exposed to sorafenib

**DOI:** 10.1002/2211-5463.12428

**Published:** 2018-04-24

**Authors:** Zhehao Dai, Haoyu Tang, Yue Pan, Junquan Chen, Yongping Li, Jun Zhu

**Affiliations:** ^1^ Department of Spine Surgery The Second Xiangya Hospital Central South University Changsha China; ^2^ Department of Minimally Invasive Orthopaedic First People's Hospital of Huaihua Jishou University of the Fourth Affiliated Hospital Huaihua China

**Keywords:** bioinformatics, functional enrichment analysis, osteosarcoma, sorafenib

## Abstract

Sorafenib is an inhibitor of a variety of tyrosine kinase receptors used to treat various cancers including hepatocellular, renal cell and thyroid carcinoma. It has been shown to change various targets associated with osteosarcoma, but the detailed mechanism remains unclear. In order to identify key genes, enriched pathways and important modules during the exposure of human osteosarcoma cells to sorafenib, data for gene expression profiles (http://www.ncbi.nlm.nih.gov/geo/query/acc.cgi?acc=GSE53155) were downloaded from the GEO database. In total, 61 differentially expressed genes (DEGs) were identified by the R bioconductor packages. Functional and enrichment analyses of DEGs were performed using the DAVID database. These revealed that DEGs were enriched in biological processes, molecular function and KEGG pathway of inflammatory immune response and angiogenesis. A protein–protein interaction network was constructed by string and visualized in cytoscape, and eight genes were selected as hubs: *IL8*,*CXCL2*,*PTGS2*,*FOS*,*CXCL1*,* C3*,*EHMT2* and *PGF*. Subsequently, only one cluster was identified by mcode, which consisted of six nodes (*CXCL1*,*CXCL2*,*PTGS2*,*FOS*,* C3* and *PGF*) and nine edges. *PGF* was the seed gene in this cluster. In conclusion, the results of this data mining and integration should help in revealing new mechanisms and targets of sorafenib in inhibiting osteosarcoma.

**Abbreviations**
AP1: activator protein‐1
BP: biological process
CC: cellular component
DEG: differentially expressed gene
ERK: extracellular signal‐regulated kinase
GO: gene ontology
MF: molecular function
mTOR: mammalian target of rapamycin
PGF: placental growth factor
PI3K: phosphatidylinositol 3‐kinase
PPI: protein–protein interaction
RANK: receptor activator for nuclear factor‐kB
RANKL: receptor activator for nuclear factor‐kB ligand
TIN: tumor infiltrating neutrophil
VEGF: vascular endothelial growth factor
VEGFR: vascular endothelial growth factor receptor

Osteosarcoma is the most common malignant cancer, primarily occurring in the bone of children and adolescents; it originates from mesenchymal stem cells and exhibits osteoblastic differentiation 1. The annual incidence rate is approximately one to three cases per million worldwide 2. With the development of surgery and chemotherapy, the 5‐year survival rate of patients with localized osteosarcoma has been greatly increased 3. However, despite improvements in osteosarcoma therapy over the past three decades, the overall survival of patients has reached a plateau and about 30–40% of patients experience progressive metastasis within 5 years after diagnosis and die 4. Therefore, exploration of novel therapeutic targets for osteosarcoma is urgent.

Previous studies have revealed that the signal transduction system plays crucial roles in osteosarcoma development, and molecules in signaling pathways are being evaluated as therapeutic targets, including those in the receptor activator for nuclear factor‐kB (RANK), Wnt, Notch, phosphatidylinositol 3‐kinase (PI3K)/Akt/mammalian target of rapamycin (mTOR) and mechanotransduction pathways 5. For instance, increased expression of RANK ligand (RANKL) in the RANKL/RANK pathway was associated with poor response of osteosarcoma patients to preoperative chemotherapy and lower cancer‐free survival 6, and specific inhibition of RANK only in osteoclasts abrogated osteosarcoma development 7. Aberrant activated Wnt/β‐catenin pathway results in β‐catenin phosphorylation and degradation inhibition, and brings about the combination of β‐catenin and lymphoid enhancer factor/T‐cell factor in cells so as to stimulate the transcription of downstream target genes in osteosarcoma 8. Deregulation of the PI3K/Akt/mTOR pathway is associated with osteosarcoma progression 9 and mTOR and PI3K are essential for osteosarcoma proliferation and survival 10.

Sorafenib is an inhibitor of a variety of tyrosine kinase receptors used to treat hepatocellular carcinoma, renal cell carcinoma and thyroid carcinoma 11 and was developed primarily as a Raf inhibitor blocking the mitogen‐activated protein kinase/extracellular signal‐regulated kinase (ERK) pathway. Furthermore, sorafenib has been shown to change a variety of other targets associated with osteosarcoma. For example, Mei *et al*. reported that sorafenib inhibits the proliferation of OS MG63 cells via changing the expression of vascular endothelial growth factor (VEGF) receptor (VEGFR) 2 and ERK, and alteration of the phosphorylation of VEGFR2, RET and mitogen‐activated protein kinase kinase 1 (MEK1) proteins 12. Pignochino *et al*. reported that sorafenib blocks osteosarcoma growth, angiogenesis and metastatis through a mechanism potentially involving the inhibition of ERK1/2, MCL‐1 and ezrin pathways 13. However, the mechanisms of response of human osteosarcoma cells exposed to sorafenib remain unclear.

In the present study, using the gene expression profile of http://www.ncbi.nlm.nih.gov/geo/query/acc.cgi?acc=GSE53155 deposited by Birgit Gallé, differentially expressed genes (DEGs) between human osteosarcoma cells (ATCC CRL‐1543™) and the same cells treated with 4 μm sorafenib were determined, and co‐expression interactions between DEGs were analyzed. Furthermore, the Database for Annotation, Visualization and Integrated Discovery (DAVID) was used to identify the significant pathways that were involved. Our study aimed to identify and explain the role of sorafenib in osteosarcoma treatment.

## Materials and methods

### Identification of differentially expressed genes from public microarray data

The public gene expression profiles of http://www.ncbi.nlm.nih.gov/geo/query/acc.cgi?acc=GSE53155 were downloaded from the Gene Expression Omnibus (GEO, http://www.ncbi.nlm.nih.gov/geo). These profiles were deposited by Birgit Gallé in 2013 and comprised three untreated replicates of human osteosarcoma cells (ATCC CRL‐1543™), and three replicates of the same cells treated with 4 μm sorafenib. The dataset was analyzed with R bioconductor packages, and raw datasets were normalized based on the preprocesscore package and the DEGs were screened out via the limma package through the cut‐off criteria of *P*‐value < 0.01 and |log_2_(fold change) |>2.

### Functional and pathway enrichment analysis

DAVID (https://david.ncifcrf.gov/) was utilized to perform functional and pathway enrichment analysis. DAVID is a systematic and integrative functional annotation tool that allows investigators to unravel the biological meaning behind large list of genes 14. Gene ontology (GO) analysis including the cellular component (CC), molecular function (MF), and biological process (BP) 15 and Kyoto Encyclopedia of Genes and Genomes (KEGG) pathway enrichment analysis 16 were carried out for the up‐regulated and down‐regulated genes separately. *P* < 0.05 was regarded as indicating statistical significance.

### Protein–protein interaction network construction and module analysis

In order to interpret the molecular mechanisms of key cellular activities in osteosarcoma cells treated with sorafenib, the online Search Tool for the Retrieval of Interacting Genes (STRING) database was used to construct a protein–protein interaction (PPI) network of the DEGs. An interaction score of not < 0.4 (medium confidence score) was considered significant and the PPI was visualized. Subsequently, the hub genes were selected according to connection degree by cytoscape software. Moreover, Molecular Complex Detection (mcode) was applied to find clusters of genes in the PPI network. ‘Degree cutoff = 2’, ‘node score cutoff = 0.2’, ‘*k*‐core = 2’ and ‘max. depth = 100’ were set as the cut‐off criteria.

## Results

### Identification of DEGs

Compared with untreated human osteosarcoma cells, a total of 61 DEGs were identified in human osteosarcoma cells treated with sorafenib, consisting of 18 up‐regulated and 43 down‐regulated genes. The top 10 up‐regulated and down‐regulated genes are listed in Table 1 (The full list of DEGs can be found in Table S1).

**Table 1:** The most significant up‐regulated and down‐regulated genes

Gene symbol	Log2(fold change)	*P*
Up‐regulated		
* MAP6*	3.2	0.00008835
* LTBP1*	2.62	0.00061335
* ADAMTSL4*	2.45	0.00007295
* LRSAM1*	2.43	0.00637533
* EHMT2*	2.41	0.00318623
* MAP6*	2.29	0.00018994
* FIG 4*	2.25	0.00108837
* REEP1*	2.16	0.00025378
* TRPC6*	2.13	0.00698107
* SEL1L3*	2.12	0.00036422
Down‐regulated		
* FOS*	−3.93	0.00000348
* KLRC2*	−3.65	0.00000474
* SCG2*	−3.38	0.00005455
* PTGS2*	−3.13	0.00016947
* STRA6*	−2.99	0.00004598
* ANGPTL4*	−2.98	0.00028895
* HES1*	−2.95	0.00042292
* STC1*	−2.93	0.00009631
* DDIT4*	−2.83	0.00040288
* CXCL1*	−2.67	0.00014383

### GO functional annotation and pathway enrichment

There were no enriched categories of GO functional annotation and pathway enrichment analysis for up‐regulated genes in DAVID.

The top 10 significant terms for down‐regulated genes are listed in Table 2. In the CC ontology, only five enriched categories satisfied the cut‐off criteria (*P* < 0.05) and we found that the majority of enriched categories were relevant to extracellular components, such as extracellular region (14 genes), extracellular space (10 genes), and blood microparticle (three genes). The other enriched categories were protein complex (five genes) and membrane (10 genes). In the BP ontology, the regulation of inflammatory immune response items constituted the majority of enriched GO categories, including inflammatory response (seven genes), immune response (seven genes), induction of positive chemotaxis (three genes), positive regulation of neutrophil chemotaxis (three genes), cell chemotaxis (three genes) and chemokine‐mediated signaling pathway (three genes). The second majority of enriched categories were associated with angiogenesis, such as angiogenesis (five genes) and positive regulation of angiogenesis (four genes). The other enriched BP GO terms contained response to lipopolysaccharide (four genes) and negative regulation of cell proliferation (five genes). In the MF ontology, only three enriched categories satisfied the cut‐off criteria, namely chemokine activity (three genes), CXCR chemokine receptor binding (two genes) and RNA polymerase II core promoter proximal region sequence‐specific DNA binding (four genes).

**Table 2:** The top 10 enriched GO terms of the down‐regulated genes

Category	GO ID and term	Count	*P*	Genes
Cellular component ontology	0005576: extracellular region	14	1.73 × 10^−5^	*CXCL1*,* HIST1H4K*,* PGF*,* C3*,* CXCL2*,* CAPZA1*,* CXCL8*,* CTSS*,* C1S*,* LAMA4*,* GLIPR1*,* EBI3*,* WFDC3*,* ANGPTL4*
	0005615: extracellular space	10	0.001789369	*CXCL1*,* C3*,* PGF*,* CXCL2*,* CXCL8*,* STC1*,* CTSS*,* EBI3*,* SCG2*,* ANGPTL4*
	0043234: protein complex	5	0.011342677	*HIST1H4K*,* PTGS2*,* SMARCE1*,* PDGFRA*,* STRA6*
	0016020: membrane	10	0.039396582	*SLC16A3*,* FOS*,* GCNT2*,* HIST1H4K*,* PGF*,* GLIPR1*,* ARFRP1*,* PDGFRA*,* METTL7A*,* IL3RA*
	0072562: blood microparticle	3	0.041879351	*C3*,* C1S*,* ANGPTL4*
Biological process ontology	0006954: inflammatory response	7	2.21 × 10^−4^	*CXCL1*,* FOS*,* PTGS2*,* C3*,* CXCL2*,* CXCL8*,* SCG2*
	0006955: immune response	7	3.88 × 10^−4^	*CXCL1*,* C3*,* CXCL2*,* RFX1*,* CXCL8*,* CTSS*,* IFI6*
	0050930: induction of positive chemotaxis	3	5.41 × 10^−4^	*PGF*,* CXCL8*,* SCG2*
	0090023: positive regulation of neutrophil chemotaxis	3	0.001179039	*CXCL1*,* CXCL2*,* CXCL8*
	0001525: angiogenesis	5	0.00172978	*PTGS2*,* PGF*,* CXCL8*,* SCG2*,* ANGPTL4*
	0045766: positive regulation of angiogenesis	4	0.002389639	*C3*,* PGF*,* CXCL8*,* ANGPTL4*
	0032496: response to lipopolysaccharide	4	0.006460182	*CXCL1*,* FOS*,* PTGS2*,* CXCL2*
	0060326: cell chemotaxis	3	0.009969718	*CXCL1*,* CXCL2*,* PDGFRA*
	0070098: chemokine‐mediated signaling pathway	3	0.011807301	*CXCL1*,* CXCL2*,* CXCL8*
	0008285: negative regulation of cell proliferation	5	0.013070326	*CXCL1*,* RARRES3*,* NDN*,* PTGS2*,* CXCL8*
Molecular function ontology	0008009: chemokine activity	3	0.005428355	*CXCL1*,* CXCL2*,* CXCL8*
	0045236: CXCR chemokine receptor binding	2	0.020082714	*CXCL1*,* CXCL2*
	0000978: RNA polymerase II core promoter proximal region sequence‐specific DNA binding	4	0.045260858	*FOS*,* NDN*,* SMARCE1*,* RFX1*

Furthermore, the KEGG pathways of down‐regulated genes were mainly involved in inflammatory immune response (Table 3), and included legionellosis (four genes), pertussis (four genes), *Salmonella* infection (four genes), leishmaniasis (three genes), Chagas disease (American trypanosomiasis) (three genes) and antigen processing and presentation (three genes). The other enriched categories comprised items related to cancer development, which included TNF signaling pathway (four genes), pathways in cancer (six genes) and PI3K–Akt signaling pathway (five genes).

**Table 3:** The top 10 enriched KEGG pathways of the down‐regulated genes

Term	Count	*P*	Genes
Legionellosis	4	6.26 × 10^−4^	*CXCL1*,* C3*,* CXCL2*,* CXCL8*
Pertussis	4	0.001630681	*FOS*,* C3*,* CXCL8*,* C1S*
*Salmonella* infection	4	0.002182574	*CXCL1*,* FOS*,* CXCL2*,* CXCL8*
TNF signaling pathway	4	0.004370617	*CXCL1*,* FOS*,* PTGS2*,* CXCL2*
Pathways in cancer	6	0.006830986	*FOS*,* LAMA4*,* PTGS2*,* PGF*,* PDGFRA*,* CXCL8*
Leishmaniasis	3	0.021059667	*FOS*,* PTGS2*,* C3*
PI3K–Akt signaling pathway	5	0.021872858	*LAMA4*,* PGF*,* PDGFRA*,* IL3RA*,* DDIT4*
Antigen processing and presentation	3	0.023923045	*KLRC2*,* KLRC3*,* CTSS*
Chagas disease (American trypanosomiasis)	3	0.042619701	*FOS*,* C3*,* CXCL8*

### PPI network construction, module analysis and hub gene selection

**Figure 1:**
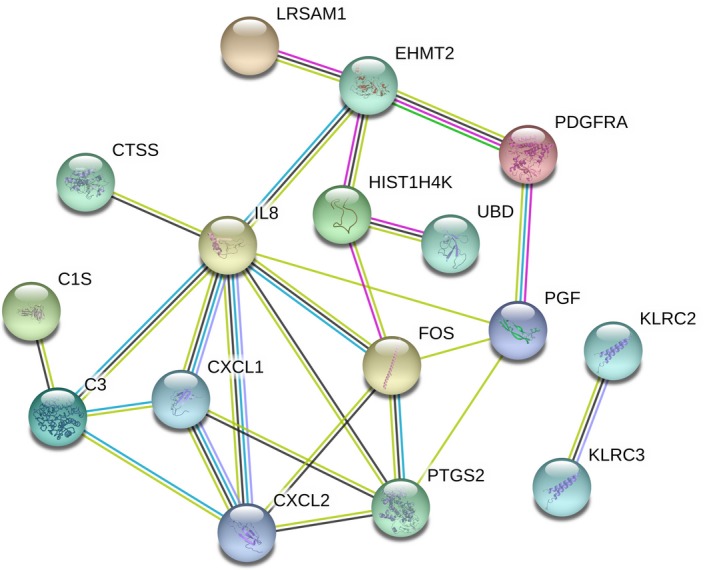
The protein–protein interaction (PPI) network for the differentially expressed genes. The nodes represent the genes and the edges represented the corresponding PPI pairs. A total of 16 genes were integrated into the network.

PPI networks were constructed on the basis of the STRING database and are displayed in Fig. 1. Most of the DEGs were disconnected in the network. When ‘Degree ≥ 4’ was set as the cut‐off criterion, eight genes in the PPI network were selected as hub genes: *IL8*,* CXCL2*,* PTGS2*,* FOS*,* CXCL1*,* C3*,* EHMT2* and *PGF*. These hub genes might play crucial roles in the mechanism of sorafenib inhibition of osteosarcoma.

**Figure 2:**
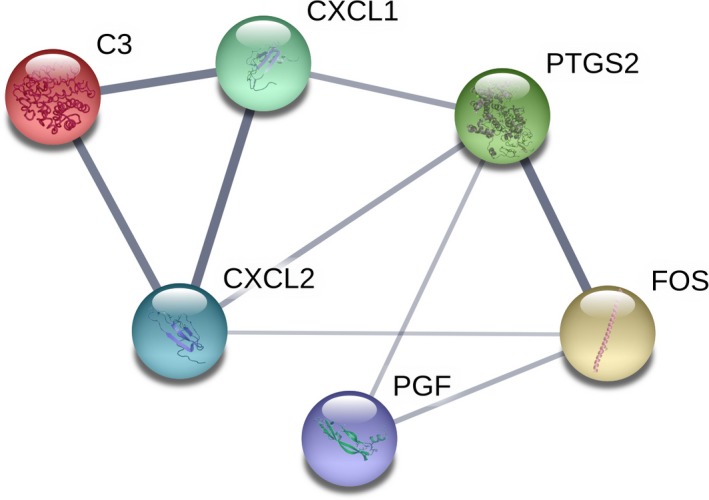
The only significant module. This module consisted of six nodes (*CXCL1*,*CXCL2*,*PTGS2*,*FOS*,* C3* and *PGF*) and nine edges, and *PGF* was the seed gene in this module.

Subsequently, only one cluster was identified from the PPI network by mcode, and consisted of six nodes (*CXCL1*,* CXCL2*,* PTGS2*,* FOS*,* C3*,* PGF*) and nine edges. Furthermore, *PGF* was identified as the seed gene in this cluster (Fig. 2).

## Discussion

Osteosarcoma is a highly malignant and very aggressive bone tumor that occurs mainly in children and adolescents and is characterized by early lung metastases and poor prognosis 1. The mechanism of sorafenib in inhibiting osteosarcoma proliferation, metastasis and invasion has not been fully reported. In order to illustrate the mechanism, a gene microarray expression profile was downloaded from the GEO database and analyzed. Compared with untreated human osteosarcoma cells, a total of 61 DEGs were identified in human osteosarcoma cells treated with sorafenib, consisting of 18 up‐regulated and 43 down‐regulated genes. *FOS*, the most regulated gene in this study, encodes c‐Fos, which is an activator protein‐1 (AP1) transcription factor. *c‐Fos* has been revealed to be overexpressed in the majority of human ostersarcomas and have an oncogenic role in osteosarcoma 17. Transgenic mice overexpressing the c‐Fos proto‐oncogene in bone develop osteosarcomas and coexpression of a c‐*jun* transgene can enhance FOS‐induced oncogenesis 18. In advanced tumors, c‐Fos–AP1 complexes were shown to induce the expression genes that are involved in angiogenesis and tumor invasiveness 19. On the other hand, knockdown of c‐*fos* inhibited the proliferation, migration and invasion of osteosarcoma cells, and promoted the apoptosis of osteosarcoma cells 20, 21. Wang *et al*. reported that miR‐101 inhibited osteosarcoma cell proliferation, migration and invasion via targeting of c‐Fos 21. Therefore, down‐regulation of c‐Fos by sorafenib is an important finding as c‐Fos acts as a significant therapeutic and prognostic biomarker. Among these DEGs, there were some genes that have not been reported in osteosarcoma, such as *KLRC2*,* SCG2*, so these might reveal the novel mechanism of sorafenib inhibition of osteosarcoma.

As was suggested by DAVID analysis, the down‐regulated genes were enriched in biological processes, molecular function and the KEGG pathway of inflammatory immune response, especially neutrophil chemotaxis. This was reasonable because tumor infiltrating neutrophils (TINs) constituted an important portion of the tumor microenvironment and contributed to the development of the tumor at multiple levels, from the remodeling of the extracellular matrix to malignant transformation, angiogenesis and modulation of other tumor‐infiltrating cells 22. TINs are striking in various solid tumors such as head and neck squamous cell carcinoma 23, non‐small cell lung cancer 24 and colorectal cancer 25, and a higher peripheral blood neutrophil count or neutrophil‐to‐lymphocyte ratio has been shown to be associated with poor survival 26. In a retrospective study performed by Yuan *et al*., 120 patients with hepatocellular carcinoma who were treated with sorafenib were enrolled and analyzed. It was reported that peripheral blood neutrophil count is a good prognostic factor for patients with hepatocellular carcinoma treated with sorafenib, and a lower peripheral blood neutrophil count was associated with a better prognosis following treatment with sorafenib therapy 27. In this study, the pathway of the inflammatory immune response, associated with DEGs such as a cluster of *CXCL* genes, was down‐regulated in osteosarcoma after the sorafenib treatment. This implies that sorafenib inhibits osteosarcoma via modulating the osteosarcoma immune microenvironment, which has not been reported before. The second majority of enriched categories was associated with angiogenesis, associated with the DEGs such as *PGF*,* CXCL8* and *ANGPTL4*. The cellular component of the GO analysis showed the majority of enriched categories were relevant to extracellular components, such as extracellular region, extracellular space and blood microparticle. The tumor microenvironment has complementary effects on the development and metastasis of osteosarcoma through extracellular secretion, alteration of phenotype type of tumor cells, immune escape and providing a proper acid–base environment for tumor cells 28.

The PPI network of DEGs provided an overview of their functional connections, of which eight hub genes were selected. Most of them were enriched in inflammatory immune response and angiogenesis. After module analysis of the PPI network, only one seed gene, *PGF*, was selected. It encodes placental growth factor (PGF), a growth factor found in the placenta that is homologous to VEGF. It was observed that binding of PGF to VEGFR1 stimulated phosphorylation of VEGFR1, induced activation of ERK1/2, PI3K, p38 and c‐Jun N‐terminal kinase and mediated their effects on the pathological conditions of vascular endothelial cell growth, inflammation and angiogenesis in several cancer cells 29, 30, 31. In osteosarcoma, a significant relationship between serum PGF level and maximum tumor size was observed 32. Therefore, down‐regulation of PGF by sorafenib might be an important mechanism by which sorafenib inhibits osteosarcoma and PGF might act as a significant therapeutic and prognostic biomarker.

Based on our study, we speculated that sorafenib may inhibit osteosarcoma by influencing the tumor immune microenvironment and angiogenesis process, and that the hub genes and seed gene play important roles. However, the benefit of sorafenib was small in clinical trials, and the progression of chemorefractory osteosarcoma was temporarily inhibited only 33, 34. The discrepancy between preclinical data and clinical data cannot be explained by our study and needs further research. Another limitation is that the mechanism of pathogenesis of the human osteosarcoma cells exposed to sorafenib needs to be elucidated through experiments *in vivo* and *in vitro*.

## Conclusion

In summary, our study identified key genes, enriched pathways and important modules during the exposure of human osteosarcoma cells to sorafenib through bioinformatics analysis. Eight hub genes and one seed gene were identified according to the PPI network. Functional and pathway enrichment analysis indicated sorafenib inhibited osteosarcoma via modulating the osteosarcoma immune microenvironment and angiogenesis. Moreover, our results could provide novel insights into the mechanisms of sorafenib treatment in osteosarcoma.

## Author contributions

HT and YP collected the data; JC and YL performed the analysis; ZD wrote the paper; JZ conceived the study. All authors read and approved the final manuscript.

## Supporting information


**Table S1.** The list of all the 61 differentially expressed genes and their fold‐changes.
